# Building on the Translational Science Benefits Model to include team science: a practical and theory-based approach to continuous quality improvement and impact evaluation for Clinical and Translational Science Award programs

**DOI:** 10.3389/fpubh.2025.1581205

**Published:** 2025-05-19

**Authors:** Kim C. Brimhall, Kayla Kuhfeldt, Darrell N. Kotton, Matthew R. Jones

**Affiliations:** ^1^Clinical and Translational Science Institute, Boston University, Boston, MA, United States; ^2^Department of Health Policy, Law, and Management, School of Public Health, Boston University, Boston, MA, United States; ^3^Center for Regenerative Medicine, Boston University and Boston Medical Center, Boston, MA, United States; ^4^The Pulmonary Center, Boston University Chobanian and Avedisian School of Medicine, Boston, MA, United States; ^5^Department of Medicine, Boston University Chobanian and Avedisian School of Medicine, Boston, MA, United States

**Keywords:** Clinical and Translational Science Award, continuous quality improvement, evaluation, Translational Science Benefits Model, logic models

## Abstract

**Introduction:**

Clinical and Translational Science Award (CTSA) programs seek to improve the quality and impact of clinical and translational science. CTSA evaluation teams implement structured, evidence-based continuous quality improvement (CQI) processes to enhance activities and outcomes, ultimately benefiting public health. The Translational Science Benefits Model (TSBM) provides a framework for assessing translational science’s health and societal impact, yet additional tools are needed to integrate CQI with impact evaluation. Addressing this gap requires combining CQI methodologies with team science approaches. Building on TSBM, CQI theories (e.g., Plan-Do-Study-Act cycles), and team science principles (e.g., inclusive leadership), we propose a theory-driven, evidence-based logic model to enhance CTSA programs. Using our TL1 Regenerative Medicine Training Program (RMTP) as a case study, we demonstrate its practical application for CTSA evaluation teams.

**Methods:**

We conducted a literature review on impact evaluation, CQI, and team science to develop a theory-based approach for CTSA evaluation teams. Using case study methodology, we analyzed RMTP data (2015–2023) through: (a) Interviews with RMTP leaders, mentors, and trainees to explore program implementation and outcomes; (b) Document analysis of program materials, meeting notes, and reports; (c) Bibliometric and policy analysis of publications, citations, and policy documents to assess impact; and (d) Surveys to capture trainees’ perspectives on program quality and leadership. This mixed-methods approach provided a comprehensive assessment of RMTP’s impact and demonstrated the utility of our team science-based approach to CQI and evaluation.

**Results:**

Our sample included RMTP directors (*N* = 2), mentors (*N* = 24), and trainees (*N* = 38). Among trainees, 68% identified as female, and 21% were from underrepresented groups in medicine. Of 34 graduates, 31 continued in regenerative medicine research. Qualitative data highlighted CQI strategies, such as embedding evaluation into advisory meetings to enhance program functioning. Inclusive leadership fostered a climate where diverse perspectives informed improvements. Quantitative and document analysis further demonstrated how RMTP activities led to positive health and societal impacts within the TSBM framework.

**Discussion:**

CTSA evaluation teams must integrate CQI and impact evaluation, yet few theory-based approaches exist. Our evaluation and CQI framework merges TSBM, CQI, and team science principles, providing a practical tool for guiding evaluation teams in continuous improvement while maximizing translational science impact.

## Introduction

Clinical and Translational Science Award (CTSA) programs have been tasked with improving the quality and impact of clinical and translational science (1–3). This involves implementing a well-structured, theory and evidence-based, continuous quality improvement (CQI) process that enhances CTSA activities and outcomes (3). Continuously striving to improve CTSA activities and outcomes helps increase the likelihood that these activities and outcomes have a beneficial impact on public health (4). The Translational Science Benefits Model (TSBM) provides a valuable framework for documenting translational science health and societal impact (5); however, more tools are needed to provide evaluation teams a theory-driven approach for simultaneously implementing CQI and impact evaluation. Although notices of funding opportunities require CTSA programs have a CQI program and measuring and evaluating CTSA public health impact, there is limited guidance on how to accomplish both CQI and public health impact evaluation (3).

Several CTSA programs have recently engaged in efforts to implement CQI processes along with impact evaluation activities. For example, Fishman and colleagues recently published their approach to CQI, highlighting the need for data collection around strategic goals to improve systems and processes (i.e., CQI) rather than solely collecting data to prove the effects of systems and processes (i.e., evaluation metrics) (3). This article provided a valuable distinction between methods and data used for CQI purposes, and those used for impact evaluation purposes. We extend and build on these efforts to provide a CQI approach that incorporates team science principles and impact evaluation using the TSBM (5). More specifically, we draw from theories on CQI (e.g., Plan-Do-Study-Act) (6–8), team science [e.g., inclusive leadership (9) and climate of inclusion (10–12)], and the TSBM framework (5) to develop a theory-driven evidence-based CQI and evaluation approach. We employ case study methodology using our TL1 Regenerative Medicine Training Program (RMTP) to demonstrate the practical application of our method and logic model. This example offers evaluation teams a concrete and adaptable framework for enhancing the quality and impact of clinical and translational science initiatives.

## Literature review

In 2011 the National Institutes of Health established the National Center for Advancing Translational Sciences (NCATS) to support CTSA programs that advance translational science. Translational science aims to address urgent public health needs through developing rapid innovations and producing effective solutions to longstanding systemic bottlenecks that slow the translational process (i.e., translating research into practice so that new treatments and health solutions reach people faster) (2). To measure the effectiveness of the overall CTSA program, NCATS launched the Common Metrics Initiative in 2015 (13). This initiative aimed to develop and implement a standardized set of Common Metrics across CTSA programs to assess the overall impact of CTSAs. These common metrics included measures on CTSA outcomes of research process efficiency (e.g., median Institutional Review Board review duration), career development (e.g., retention and diversity of CTSA scholars/trainees in clinical and translational research), and scientific productivity (e.g., pilot and grant funding awards, publications) (5). While these metrics may provide insight into CTSA operational effectiveness, they are less informative on how CTSA activities can be improved and CTSA’s long-term impact on translational science (5). In response, CTSA programs have been recently tasked with ensuring their activities and outcomes effectively lead to meaningful public health benefits (3, 5), suggesting CTSA evaluators need to implement CQI of CTSA activities and evaluate CTSA outcomes for public health impact. Research demonstrates the positive effects of implementing CQI processes as a way to enhance the efficiency and effectiveness of various CTSA activities (3), with the goal of increasing the likelihood these activities have a beneficial impact on societal and public health.

### Translational Science Benefits Model

The Translational Science Benefits Model (TSBM) provides a valuable framework for documenting translational science health and societal impact (5). This relatively new framework identifies four main domains of how clinical and translational science can benefit health and society: clinical and medical, community and public health, economic, and policy and legislative. These domains provide a way of organizing how clinical and translational science can have an impact on public health and well-being (14). The clinical and medical domain refers to procedures, guidelines, tools, or products that were developed from clinical and translational research and implemented in clinical and/or medical practice. The community and public health benefits domain refers to the enhancement of health care, community, and/or population well-being as a result of clinical and translational research (e.g., improvements in health activities and products, health care characteristics, and/or community health promotion). Economic benefits of clinical and translational research can refer to developed commercial products, financial savings and benefits, and increased economic mobility of trainees and scholars. The fourth main benefit domain refers to policy and legislative benefits, including the ability of translational science to influence advisory activities and the decision-making process of organizational or public policies, legislation, or governmental standards. This can include how translational research informs policymaking and is used in formal adoption of policies and legislation, such as organizational guidelines and internal agency decisions as well as formal laws or rules enacted by governmental bodies (5, 14).

The TSBM framework can be a valuable tool for providing a common language around tracking the public health impact of clinical and translational science; however, more tools are needed to provide evaluation teams a theory-driven approach for simultaneously implementing CQI and impact evaluation. Given that CTSA programs have been tasked with accomplishing both, CQI and impact evaluation, we strived to create a theory-based framework that brought together CQI, impact evaluation using the TSBM, and team science.

### Continuous quality improvement

Table 1 provides a summary of common CQI approaches. Rooted in the scientific method, CQI methods have been used to iteratively improve health care (8, 15) and more recently encouraged to enhance CTSA efforts toward translational science (1–3). The Plan-Do-Study/Check-Act (PDSA/PDCA) cycle of CQI is one of the most widely used methods within health care and considered a key foundational approach to quality improvement (6–8). The first stage in the PDSA/PDCA is “plan” or the prediction/hypothesis of testing a particular change. The “do” part of this cycle refers to implementing the planned change, whereas the “study” or “check” portion of the cycle refers to analyzing the effects of the change (hypothesis testing). The “act” part of the cycle generally refers to reaching a conclusion with another prediction of what to do next in the “plan” stage of the PDSA/PDCA (16).

**Table 1:** Overview of continuous quality improvement (CQI) models.

CQI model	Key steps		Unique features
PDSA/PDCA	Plan Do Study Check/Act		Foundational model for iterative continuous improvement.
FOCUS-PDCA	Find process Organize team Clarify process Understand variations Select improvements	then apply: 6. Plan 7. Do 8. Study 9. Check/Act	Extension of PDSA/PDCA to enhance process efficiency.
FADE	Focus (identify problem) Analyze (data analysis) Develop (solutions) Execute (implement plan)		Linear process for problem identification, analysis, and execution of solutions; Ideal where clear problems exists & one-time solutions needed.
Logic framework	Identify improvement areas Conduct root cause analysis Create problem & objective trees Formulate framework Execute projects		Logical reasoning for structured problem-solving; Uses structured analysis tools like problem & objective trees.
Breakthrough series	Team collaborative learning sessions Share experiences Discuss progress		Emphasizes team learning, knowledge sharing, & cross-organizational collaboration.
Lean 5S	Sort Set/Straighten Shine Standardize Sustain		Focuses on workplace organization, efficiency, & reducing waste.
Kaizen	Continuous small improvements Problem-solving Employee empowerment		Encourages incremental, practical, low-cost changes & process discipline.
Lean Six Sigma DMAIC/DMADV	Define Measure Analyze Improve Control	Define Measure Analyze Design Verify	Data-driven process optimization; Reduces variability & waste while ensuring process stability (DMAIC used for current processes; DMADV used for new processes).
5C Cyclic Model	Consultation Collection Consideration Collaboration Celebration		Community-driven quality improvement; Designed for volunteer healthcare services in Aboriginal communities.
WE-CQI	Collaborative planning Shared action Team reflection & learning		Combines team science, TSBM, & PDSA/ PDCA, Kaizen, Breakthrough Series, & Logic Modeling into a simplified 3-step approach.

A successful CQI process is learning (16). Learning may come from achieving quality improvement goals (the tested change in a CQI approach worked). Learning can also come when quality improvement goals are not achieved, often uncovering unanticipated constraints that need to be addressed and/or identifying new problems central to the originally identified challenge. In other words, a well-conducted CQI approach promises learning, not that specific quality improvement goals will achieve their desired outcomes (17). The task of CQI methods, such as the PDSA/PDCA, are to translate ideas for improvement into action, evaluate that action to encourage learning, and then ultimately improve the quality of what’s done. Several CQI frameworks have been developed to expand upon the PDSA/PDCA approach.

The FOCUS-PDCA cycle enhances the PDSA/PDCA process by adding steps to find and improve a specific process, organize a knowledgeable team, clarify the selected process, understand variations in the selected process, and choose possible process improvements (18). The FADE approach is more of a linear CQI process that involves identifying a problem, understanding it through data analysis, developing solutions, and then implementing the solution plan (18). Similarly, lean CQI approaches follow more linear and data-driven steps (e.g., value stream mapping and root cause analysis) to quality improvement. The Lean 5S approach focuses on five ordered steps (sort, set/straighten, shine, standardize, sustain) to help reduce workplace waste by enhancing organization and efficiency (18, 19). The Kaizen approach focuses on more incremental and practical improvements through empowering employees to problem-solve, using data to drive change, acknowledging process defects, reducing variability and waste, and maintaining a disciplined workplace. Lean Six Sigma involves five steps that define (D) and measure (M) the problem, analyze (A) root causes, develop (D) or improve (I) solutions, and control (C) or verify (V) process stability (19). DMAIC is used for current process improvement whereas DMADV is used for developing new processes for improvement. Another CQI approach which incorporates data-driven methods and root cause analysis is the Logic Framework. This approach involves brainstorming to identify improvement areas, conducting root cause analysis to develop a problem tree, logical reasoning to create an objective tree, formulating the framework, and executing improvement projects (20). A more collaborative CQI framework includes the Breakthrough Series, which requires CQI teams to meet in quarterly collaborative learning sessions, share learning experiences, and continue discussion by telephone and cross-site visits to strengthen learning and idea exchange (18). A collaborative community-driven CQI approach is the 5 C-cyclic model (consultation, collection, consideration, collaboration, and celebration). This approach was originally designed for volunteer dental services in Aboriginal communities to improve quality of care based on community needs (18).

Many of the CQI frameworks were developed for specific organizational or programmatic quality improvement purposes and may not yield the flexibility needed for quality improvement in more complex multi-team systems, like CTSAs. For example, some of the most common challenges to CQI efforts involve individual resistance to change, discomfort with inter-professional collaboration, and failing to create a positive organizational climate conducive to CQI. Literature indicates possible solutions to common CQI challenges, such as qualified leadership that can foster collaborative workplace cultures (18), however, more research is needed on specific leadership approaches and organizational climates that support and engender CQI, particularly in complex interdisciplinary multi-team systems. Thus, using evidence-based and theory-driven team science approaches we developed a framework for CQI and evaluation to help mitigate common barriers to successful CQI. Our framework incorporates PDSA/PDCA’s iterative improvement cycle, aligns with the Breakthrough Series and Kaizen approaches by emphasizing collaborative learning, team-based reflection, and valuing incremental and practical improvements, and uses the Logic Framework by incorporating logic models to support structured problem-solving. The unique and novel contributions of our framework is that it simplifies these multiple CQI approaches, incorporates principles of team science, and applies the TSBM into a streamlined three-step process (collaborative planning, shared action, and team reflection/learning). Unlike CQI models with more rigid and linear steps (e.g., FADE, DMAIC), our approach is designed to be flexible and adaptive to dynamic team environments, such as multi-team complex systems like CTSAs. In essence, our framework applies team science principles, integrating collaboration, knowledge sharing, and iterative learning into a simplified yet comprehensive improvement model designed to promote TSBM impacts.

### Team science

CTSA programs are multi-team systems wherein multiple groups of individuals (often diverse and multidisciplinary team members) must work together to accomplish CTSA objectives. This means individuals must work effectively within their respective teams and across multiple teams within the CTSA to accomplish program objectives. This requires a novel approach to quality improvement and evaluation methods that incorporates team science. Team science is a translational science core principle and one of NCATS’s strategic goals (21, 22), as it focuses on best practices for engaging multidisciplinary team members around shared objectives, such as implementing quality improvement and impact evaluation processes. Below we describe our evaluation and CQI approach grounded in team science theories that strive to create an inclusive organizational climate. Inclusion has been shown to be an effective organizational management and team science approach for creating environments wherein teams can openly share ideas with one another around complex challenges (23, 24) and generate new methods for quality improvement (9).

## Theory-based framework

### Wisdom-driven evaluation and continuous quality improvement

**Figure 1:**
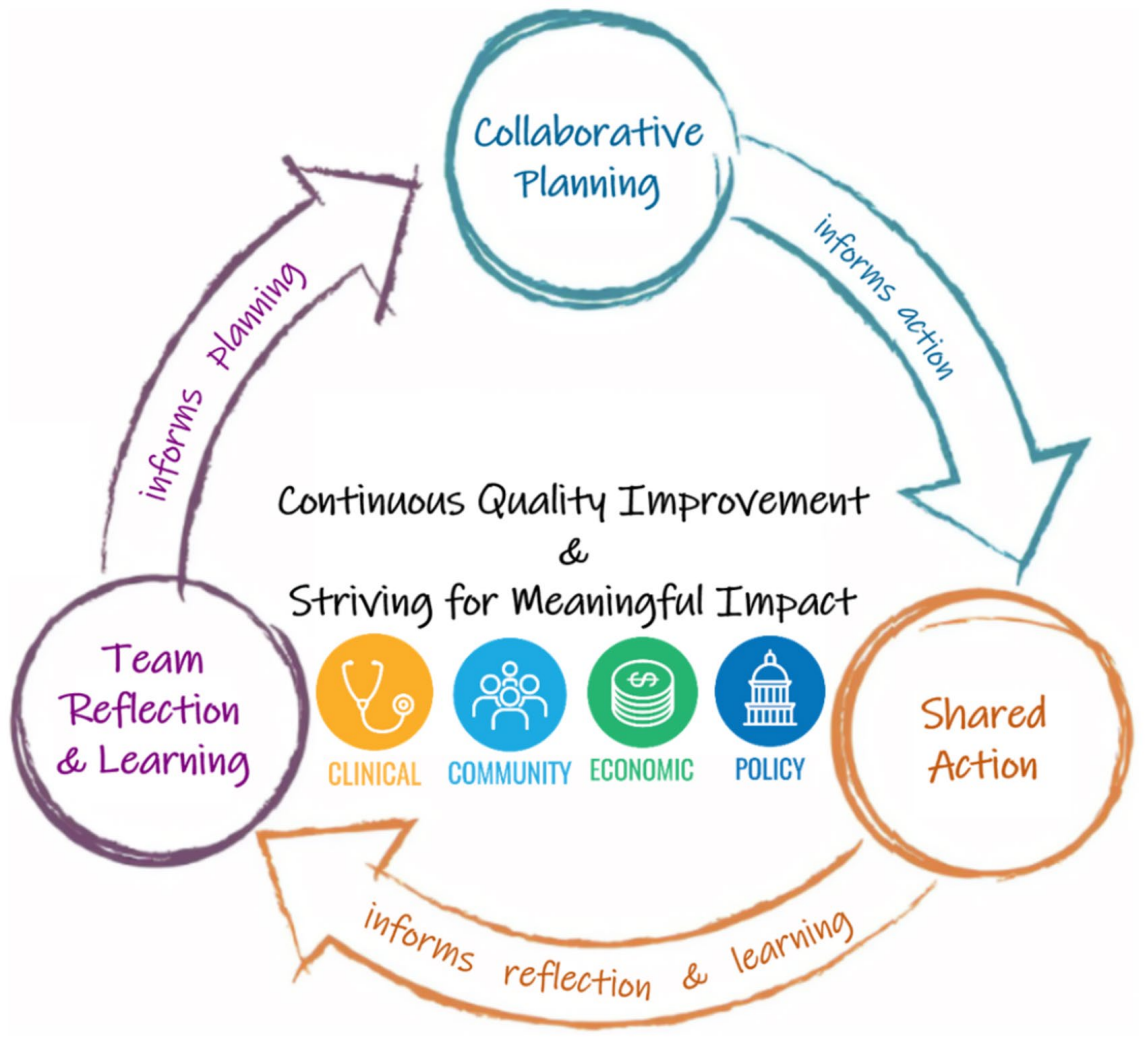
Wisdom-driven evaluation and continuous quality improvement (WE-CQI). TSBM icons: The Translational Science Benefits Model and Translating for Impact Toolkit^©^ 2017–2023, created by the Institute of Clinical and Translational Sciences at Washington University in St. Louis and available at *translationalsciencebenefitsmodel.wustl.edu*, is licensed under *Attribution-NonCommercial-ShareAlike 4.0 International (CC BY-NC-SA 4.0)*.

Drawing from theories of team science and several CQI frameworks, we develop a team-based quality improvement process designed to provide a theory-driven approach to CQI and impact evaluation for CTSA programs. More specifically, we draw on team science theories of inclusive leadership (9) and climate for inclusion (10–12) as best practices for implementing wisdom-driven evaluation and CQI (WE-CQI). We define WE-CQI as the ability to use collective knowledge and experiences to make shared decisions on measuring the quality and impact of something, including CQI efforts and evaluation of these efforts. There are three phases: collaborative planning, shared action, and team reflection and learning (see Figure 1).

#### Collaborative planning

The first phase, collaborative planning, involves engaging a representative group of team members who are involved in accomplishing the specific CTSA program objective. Sometimes this requires bringing together team members from the same program who focus on a specific CTSA objective and sometimes this involves bringing together multiple teams from different programs within a CTSA that partner to accomplish the CTSA objective. The CTSA evaluation team facilitates the WE-CQI meeting(s) focused on collaborative planning and decision-making around best practices for accomplishing the objective and generating ideas for quality improvement. During the collaborative planning phase, evaluation team members strive to use principals of inclusive leadership. Leader inclusiveness has been defined as the “words and deeds by a leader or leaders that indicate an invitation and appreciation for others’ contribution” (9). When leaders engage all team members by seeking input from others in decision making, encouraging everyone to take initiative in organizational processes (e.g., quality improvement and evaluation processes), and expressing equal value for the contributions of others, individual participation and engagement efforts increase (25). This type of leader inclusiveness goes beyond simply sharing decision making; it strives to foster intergroup contact by helping members feel valued and appreciated for their unique perspectives, regardless of individual job positions within the CTSA or personal educational backgrounds. The leader’s ability to encourage the participation of all members and expressing value for their unique perspectives aligns with the theoretical foundation for creating an inclusive climate (10–12, 26).

Optimal distinctiveness theory suggests that individuals feel included when they are valued for their uniqueness while also experiencing a sense of belonging within the group (12, 26). When leaders demonstrate inclusiveness by acknowledging and appreciating team members’ unique perspectives, they reinforce the value of individual uniqueness. For example, when CTSA leaders or evaluators seek feedback and publicly recognize a team member’s contributions, they signal that the team member is a valued part of the group (27, 28). This, in turn, encourages other CTSA members to appreciate their contributions, fostering a stronger sense of belonging (26). Leader inclusiveness ensures that each team member feels valued, and by fostering both uniqueness and belonging, it creates an environment where individuals feel comfortable sharing their ideas with one another (29, 30).

Leader inclusiveness has been linked to increased psychological safety, which enables team members to take interpersonal risks, such as speaking up and sharing their ideas, experiences, and knowledge (31). This open exchange is essential during the collaborative planning phase of WE-CQI meetings, as team members must feel valued and engaged in developing the collaborative plan. However, if team members believe the perspectives they shared were not valued (not acknowledged, discussed, or incorporated), the quality improvement plan fails to reach a level of collaboration and instead may be viewed by team members as a performative exercise (an illusion of inclusion). For a collaborative plan to truly be collaborative, team members need feel a sense of empowerment and responsibility for the creation of the plan (team members are full partners in the plan’s creation).

#### Shared action

Once the collaborative plan has been created, team members responsible for accomplishing the CTSA objective share responsibility in the implementation of the collaborative plan. Leadership research suggests a narrow to medium span of control for CTSA leaders yields optimal results for team member satisfaction and performance (32, 33), suggesting CTSA leaders have ideally up to 10 team members they supervise (34). Thus, we have designed a proactive leadership structure to ensure CTSA program team members feel supported in the implementation of the collaborative plan. This structure includes quarterly WE-CQI meetings with the CTSA evaluation team and members of the CTSA program involved in the collaborative plan for quality improvement and evaluation of the specific CTSA objective. The purpose of the quarterly WE-CQI meetings is to provide consistent time to review the collaborative plan implementation and problem solve unanticipated barriers or challenges to implementation (these WE-CQI meetings are also scheduled as needed if major unanticipated barriers arise). In addition to quarterly WE-CQI meetings, the evaluation team holds bi-weekly WE-CQI meetings with program staff and managers. Each CTSA program has at least one manager (some programs have multiple managers depending on team size to ensure an optimal span of control) who are primarily responsible for the day-to-day implementation of program activities. These bi-weekly WE-CQI meetings are designed to be proactive in nature by providing training for managers in best practices for quality improvement and evaluation activities, reinforce a culture of inclusion and partnership in the shared action of the collaborative plan, and reserve time for staff to check-in and problem solve for minor unanticipated challenges with collaborative plan implementation [major barriers or challenges are brought to the quarterly (or as needed) WE-CQI meetings where all program team members are present].

#### Team reflection and learning

Once the collaborative plan has been implemented through shared action from members of the CSTA program, the evaluation team facilitates team reflection and learning through quarterly WE-CQI meetings. Similar to the collaborative planning phase, principles of inclusive leadership (9, 25) and inclusion (10–12) are used to facilitate team reflection on the implementation of the collaborative plan and evaluation of the tested quality improvement change implemented. Through open team dialogue, team members reflect on data collected on the implementation process, quality improvement change, and ultimately TSBM potential (or realized) impacts. A climate of psychological safety (35) is critical for successful team reflection and learning (36), particularly when discussing potentially sensitive topics, such as when quality improvement efforts fail to yield anticipated results. Inclusive leadership and climate for inclusion are critical antecedents to (25, 36) open and honest communication which enables learning from quality improvement processes and creating new avenues to further improve (31, 36). Thus, an open and honest discussion among all team members is needed to promote meaningful reflection of quality improvement efforts, determine whether continued changes are needed to effectively meet CTSA objectives, and evaluate the health and societal impact of CTSA activities. The team reflection and learning phase then informs the next collaborative planning phase in the WE-CQI framework for CQI and striving for meaningful impact.

## Application and case study

**Figure 2:**
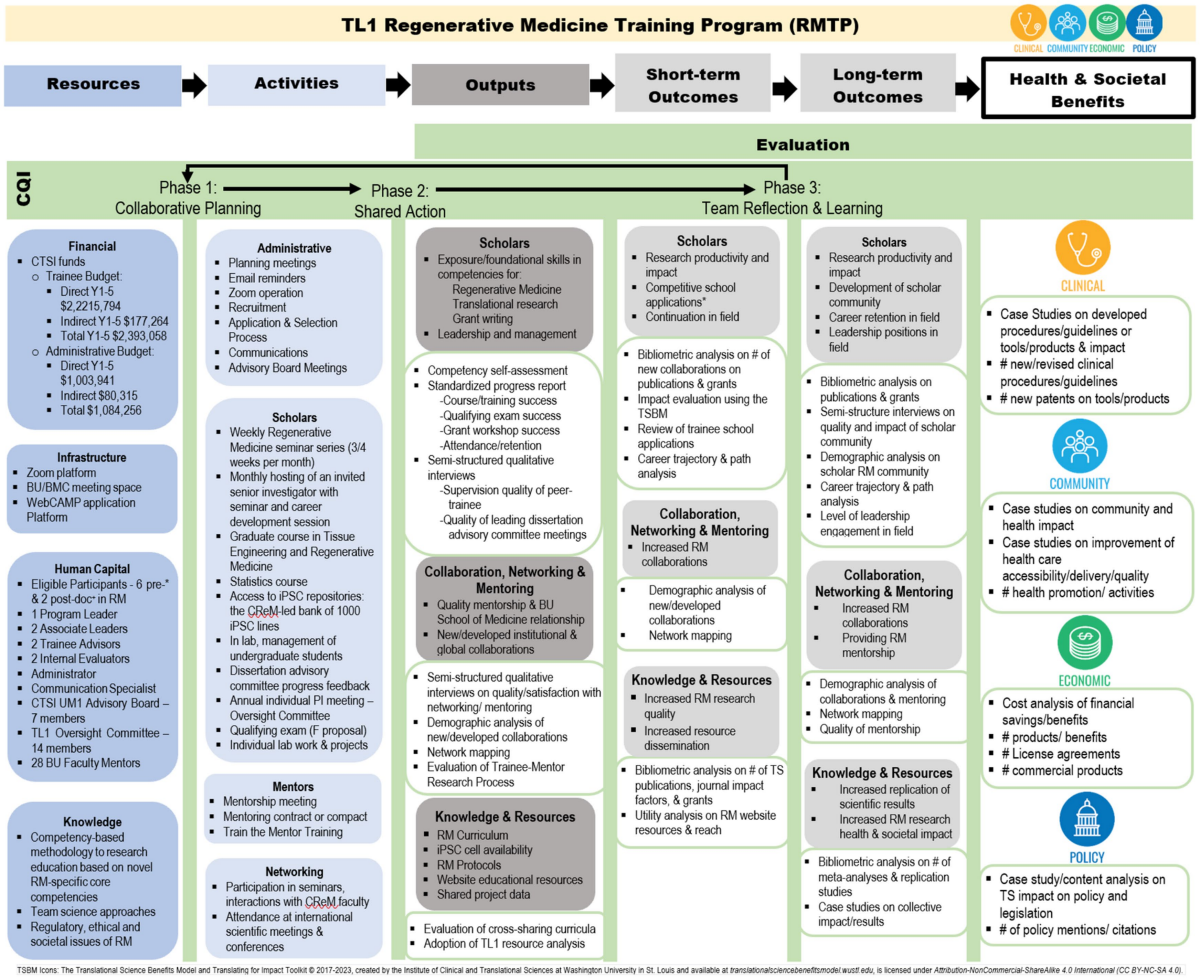
WE-CQI logic model example.

Building on the TSBM framework (5) we develop a logic model that incorporates our theory-driven and team science-based approach to CQI and impact evaluation. We utilize a case study approach to demonstrate how our logic model can be applied with our TL1 Regenerative Medicine Training Program (RMTP), providing a practical approach for CTSA evaluation teams striving to improve the quality and impact of clinical and translational science (see Figure 2; Table 2).

**Table 2:** Examples of RMTP CQI, evaluation, and TSBM impact activities.

Level	Example of CQI activities	Example of evaluation activities	Example of impact evaluation	
Trainee	Competency assessment Survey on mentorship quality End-of-training satisfaction survey Training implementation acceptability, adoption, feasibility, & fidelity	Academic/research progress: trainee & mentor reporting on trainee academic/research progress, competencies, development of translational scientist characteristics & leadership skills Bibliometric analysis during & after program; research productivity, influence, impact, co-author collaborations, & career pathway tracking	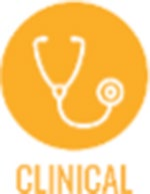	Development of clinical guidelines/ procedures Case studies on implementation/impact of clinical guidelines/ procedures
Mentor	Mentorship behaviors & quality survey Mentor support satisfaction survey Mentor training implementation acceptability, adoption, feasibility, & fidelity	Bibliometric analysis during program & mentorship: research & scholarly productivity, influence, impact, & network growth analysis	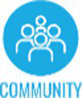	Development of translational science & community/health education resources
Program	Regular review of data, processes & outcomes for overall CQI: collaboratively plan (identify & operationalize strategies for improvement), shared action (implement strategies & collect data), & team reflection & learning (assess impact & decide to adopt, adapt & test for a second cycle, or abandon strategies)	Fidelity & effectiveness of trainee & mentor recruitment: demographic analysis (trainees & mentors); assessment of recruitment activities/challenges/modifications/selection Dissemination & adoption of research/training resources: Tracking protocol downloads, iPSC vials shipped, stem cell & gene editing repositories, website visits, training curricula & resources Pre-& post trainee assessment of translational science competencies, characteristics, & knowledge	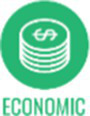	New license agreements/patents on developed intellectual property initiated by RMTP trainees/mentors/program
			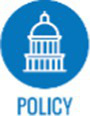	Influence on policy & legislation (measured using Overton policy analysis)

TSBM icons: The Translational Science Benefits Model and Translating for Impact Toolkit^©^ 2017–2023, created by the Institute of Clinical and Translational Sciences at Washington University in St. Louis and available at *translationalsciencebenefitsmodel.wustl.edu*, is licensed under *Attribution-NonCommercial-ShareAlike 4.0 International (CC BY-NC-SA 4.0)*.

### WE-CQI logic model

Logic modeling refers to the process through which evaluators discern, represent and utilize program theory to design and implement each stage of evaluation (37). As a standard evaluation practice, logic modeling can enhance alignment and efficiency between data collection for CQI and evaluation and program activities and objectives (37). In the WE-CQI framework, logic modeling is used as part of the collaborative planning phase to co-design how CQI and evaluation activities are embedded within the CTSA program objectives. Members of the CTSA program and evaluators co-create the WE-CQI logic model to: (a) represent how program resources, activities, and outputs lead to short-and long-term outcomes and TSBM impacts; (b) design program CQI and evaluation activities; and (c) clarify shared roles and responsibilities of CTSA team members in the implementation of program activities, CQI, and evaluation. It is important to note that while all TSBM domains (i.e., clinical, community, economic, and policy) are represented in the WE-CQI logic model, not all CTSA programs will have equal impacts in each of the TSBM domains. For example, some CTSA programs, like the Community Engagement program may have more TSBM impacts in the community domain as opposed to the economic domain, and the RMTP program may have more impacts in the TSBM economic domain relative to the community domain. The overall goal of developing WE-CQI logic models for each CTSA program is to highlight which programs lend to specific TSBM impacts, with the ultimate goal of the entire CTSA having impacts in each TSBM domain.

To illustrate the WE-CQI’s logic model application, we use the RMTP program as a case study. First, we listed all the resources (CTSA and other) that support the RMTP in the far-left column. Resources directly influence the activities the RMTP is able to perform and thus RMTP activities are listed in column two. Activities are then linked to anticipated outputs along with how these outputs are being measured (appear directly under each output description in the output column). Outputs are linked to short-term outcomes along with how short-term outcomes are being measured (appear directly under each short-term description in the short-term outcomes column). Short-term outcomes are then linked to long-term outcomes along with their associated methods which appear directly under each long-term description. Finally, long-term outcomes are linked to down-stream health and societal benefits (and how these are measured) following the TSBM framework that includes clinical, community, economic, and policy benefits. Given RMTP is a training program embedded within a CTSA, it is important to note that some short-term and long-term outcomes, such as research conducted in collaboration with RMPT leaders, CTSA members and trainees may lead to immediate and direct health and societal impacts (e.g., new clinical and/or procedural guidelines for treating and studying infectious disease), as well as more long-term and down-stream TSBM impacts (e.g., the development of safe and cost-effective treatment options).

#### Collaborative planning

During the collaborative planning phase of our WE-CQI approach we examine current resources and activities that support the RMTP. This provides a foundation for understanding the current resource structure and activities being performed by the RMTP, which enables realistic planning for areas of improvement. The collaborative planning phase is placed in-between the resources and activities columns of the logic model given the high possibility of needing to allocate resources and/or adjust or re-envision activities to support planned WE-CQI efforts. It is important to note however, when collaboratively planning for quality improvement and impact evaluation, the entire logic model is reviewed given that CQI efforts may be identified throughout the logic model. For example, reviewing outputs, short-or long-term outcomes, and health and societal benefits, may uncover areas needing improvement which often requires changes/adjustments in resources and activities.

#### Shared action

The shared action phase of the WE-CQI framework is visually placed in-between the activities and outputs columns of the logic model to represent how RMTP activities are often partnerships across the CTSA and other teams, and how implemented WE-CQI initiatives during the collaborative planning phase become shared actions by RMTP and evaluation team members. In other words, quality improvement efforts developed in the collaborative planning phase become a shared responsibility among team members to implement the developed quality improvement plan (i.e., shared action).

#### Team reflection and learning

During the team reflection and learning phase of the WE-CQI framework, all members of the RMTP and CTSA evaluation team have an opportunity to reflect on quality improvement and evaluation data collected along with lived experiences of team members during the implementation of quality improvement efforts. This phase involves a comprehensive review of data collected from RMTP activity outputs, short-and long-term outcomes, and health and societal benefits, providing a holistic assessment of the program’s progress and impact. During this reflection process, the team critically examines whether the CQI strategies developed in the collaborative planning phase effectively enhanced the RMTP objectives and overall impact. Integrating both quantitative and qualitative insights from team members’ experiences fosters a comprehensive and meaningful reflection process. This approach enables the identification of successes, challenges, and areas for improvement. This iterative review not only strengthens the RMTP but also supports alignment with the TSMB by emphasizing impacts across clinical, community, economic, and policy domains. The reflection and learning phase concludes with the development of new strategies and priorities, which directly inform subsequent collaborative planning and CQI efforts, ensuring a cycle of continuous enhancement and alignment with translational science goals.

### Case study methods

To illustrate our WE-CQI framework, case study methods on the RMTP were used to provide a practical application of the logic model. Case studies offer evidence about causal inference and program implementation and are widely recognized as an invaluable resource for understanding the dynamic influence of context on interventions, such as the RMTP training program. For example, case studies directly inform assessments of where, when, how and for whom the RMPT training program might be successfully implemented, by specifying the necessary conditions under which the program may have effects and to consolidate learning on how interdependencies and unpredictability can be managed to achieve and sustain desired effects (38). Data were collected from 2015 to 2023 using a mixed-methods approach that included semi-structured qualitative interviews with RMTP leaders, mentors, and trainees, document, bibliometric, and policy analysis, and quantitative surveys.

Semi-structured qualitative interview guides of open-ended questions were co-designed with the RMTP leaders and CTSA evaluators to assess quality and satisfaction with RMTP training and mentoring. Interview guides were reviewed yearly by RMTP leaders and CTSA evaluators and revised if needed based on participant feedback. Each RMTP participant trainee in each cohort were interviewed twice throughout their training program, a mid-point interview (typically at the end of Year 1) and a final exit interview at the end of their appointment. To protect trainee confidentiality and promote open and honest feedback, CTSA evaluators conducted interviews individually with trainees in a private meeting space and/or via Zoom. Interviews were transcribed by CTSA evaluators, and all personal identifying information of trainees were removed from the data. De-identified data was aggregated such that all participant responses were combined by interview question to ensure participant anonymity. Using constant comparative methods, qualitative data was thematically analyzed by two independent CTSA evaluators (39–42). Differences in emerged themes and codes were discussed until consensus was reached (interrater reliability of 90% or better). Qualitative data was used to help contextualize quantitative data (i.e., survey, bibliometric, policy and document analyses) and final summary reports were created to provide RMPT scholars, mentors, and leaders evidence-based feedback for guiding program enhancements.

Two separate quantitative surveys were co-designed by CTSA evaluators and RMTP leaders. A trainee survey (to be completed by the RMPT trainee) was used to assess the trainee’s perceptions of quality and satisfaction with RMTP training and mentoring as well as their progress, challenges, and accomplishments. A separate mentor survey (to be completed by the trainee’s mentor) was used to assess mentor perceptions of their trainee’s progress, challenges, and accomplishments. Trainee and mentor survey results were matched by trainee-mentor pairs to examine possible discrepancies in trainee and mentor perceptions of the trainee’s progress, challenges, and accomplishments. These surveys were distributed yearly to both the trainee and the trainee’s mentor and sent through a secure standardized progress report application in WebCAMP (i.e., analytic tool created by Weill Cornell Medicine’s Clinical and Translational Science Center in 2014). The WebCAMP software stores both administrative and evaluation data linked to RMTP training program implementation. It is centralized for use by our CTSA evaluator team who ensures data quality and reporting to the CTSA leaders and governance groups such as External Advisory Boards and internal oversight committees.

Document analysis included thematic reviews of internal standardized action plans (used to measure and assess trainee progress and individualized trainee programmatic changes over time); bibliometric analyses using a variety of external software analysis tools (e.g., Dimensions, BU Profiles, and iCite) to measure and assess research productivity, influence, and impact over time; and policy analyses conducted using external Overton policy analysis software (43). Publications that cite the RMTP program and all publications written by RMTP trainees were searched via the Overton tool, which shows any policy documents or policy mentions that utilize the specific publication. These policy documents are then aggregated and displayed in an overall report, highlighting policy sources by location (e.g., country), organization type, funder of cited research, publication date, and policy subject areas. For the purposes of the case study and application of the WE-CQI RMTP logic model, all data from document analysis, surveys, and interviews were aggregated by topic and theme (e.g., RMTP resources, activities, outputs, short-and long-term outcomes, and TSBM impacts). Specific examples of CQI efforts that emerged from the data were highlighted for more in-depth illustration of the WE-CQI framework.

### Case study results

The sample includes RMTP Program Directors and CTSA members (*n* = 2), mentors (*n* = 24), and trainees (*n* = 38). Of the RMTP trainees, 66% self-identify as female and 21% self-identify being from a National Institutes of Health (NIH) defined under-represented racial and/or ethnic demographic group in medicine (44). 34 RMTP trainees have completed the two-year RMTP program, with 31 trainees remaining in regenerative medicine research and 3 trainees in healthcare consulting, medical writing, and health system clearance coordinating careers. Qualitative data from RMTP leaders revealed critical CQI processes that led to successful program implementation. This includes strategies that disentangle roles and responsibilities of CTSA team members and thoughtful approaches for how CQI and impact evaluation activities can be embedded in the logic model and throughout the program. For example, case study findings revealed a CQI process that RMTP leaders implemented to ensure timely reflection on program functioning and seek opportunities for program improvement from diverse perspectives: RMTP leaders implemented evaluation as a standing agenda item in their internal advisory committee meetings. This created dedicated time to review and reflect on program evaluation data (e.g., program activities, trainee/mentor feedback, barriers and challenges to trainee progress) and to discuss opportunities for program enhancement with members of the evaluation team and internal advisory committee.

### Case study CQI & TSBM impact examples

The RMTP places a strong emphasis on cross-disciplinary collaboration and communication skills, centered in team science principles, such as inclusive leadership and fostering a climate for inclusion where diverse perspectives are actively shared and valued. Results from the CQI process, guided by these principles, have driven several programmatic changes that improved implementation and outcomes for RMTP trainees. Likewise, results from quantitative data and document and policy analysis demonstrate impact evaluation activities embedded within the logic model that connects how RMTP resources, activities, outputs, and outcomes led to positive health and societal impacts within the TSBM framework.

#### CQI building a sense of community

Trainee feedback from the Fall 2016 mid-program interviews (*n* = 8) revealed that trainees felt a lack of community with their peers. In response, the RMTP Program Director initiated two annual luncheons to facilitate peer interactions and build connections among trainees. By the 2018 exit interviews (*n* = 4), feedback indicated a strengthened sense of community as a result of this initiative. However, with the COVID-19 Pandemic in 2020, trainees again were impacted with their sense of community due to the need to self-isolate and not gathering in social or work settings. In the 2022 mid-point interviews (*n* = 8), majority of the interviewed trainees (75%) indicated the COVID-19 Pandemic influenced their training, including delays in lab training, feelings of isolation, loss of opportunities, and loss of mentorship time and opportunities. Recent interviews in 2023 (*n* = 8), indicated the majority of trainees (75%) believed the COVID-19 Pandemic was still having an impact due to the lack of availability of additional trainings from staffing shortages, limited resources due to supply chain issues, delays in graduation and project timelines, and lack of one-on-one mentorship during the pandemic. Nevertheless, trainees expressed a renewed sense of connection as they resumed attending seminars at the Boston University’s Center for Regenerative Medicine (CReM), working collaboratively in shared laboratory spaces, and attending meetings with mentors.

To illustrate how CQI and impact evaluation examples from the case study align with the WE-CQI logic model—and to further demonstrate its application—we describe in more detail below how the *CQI Building a Sense of Community* example maps onto the WE-CQI logic model. In the Resources column of the WE-CQI logic model, financial (e.g., administrative support in coordinating additional trainings), infrastructure (e.g., integrating zoom as a platform for creating community and offering mentoring) and human capital (e.g., availability of mentors) resources were used to influence the types of activities supported in building a sense of community. In the Activities column of the WE-CQI logic model, administrative (e.g., coordinating annual luncheons for peer and mentor networking), scholar (e.g., training seminars), mentors (e.g., one-on-one mentorship meetings with trainees), and networking (e.g., CReM seminars) were particular activities related to building a sense of community for trainees. The Outputs column of the WE-CQI logic model displays outputs of RMPT activities as well as how outputs are being evaluated for CQI and impact [scholars (e.g., qualitative interviews on trainee satisfaction with mentorship), collaboration, networking and mentoring (e.g., examination of survey data on discrepancies between trainee-mentor perceptions on trainee research progress, barriers, and accomplishments), knowledge and resources (e.g., examination of trainee attendance and engagement with RMPT curriculum trainings and CReM networking seminars)]. This information helped uncover whether implemented CQI efforts, such as establishing annual luncheons with trainees, were effective in building a sense of community for trainees. Short-Term and Long-Term Outcomes are also represented in the WE-CQI logic model as well as CQI and evaluation methods for assessing these outcomes. This further helped evaluate the impact of implemented CQI efforts designed to build a stronger sense of community among trainees, e.g., short-term outcomes of increasing available mentorship through increased one-on-one mentor meetings and CReM networking seminars resulted in broader mentorship networks and long-term outcomes of increased training opportunities and collaborations on research publications. Research collaborations and mentored research publications are followed to examine the down-stream TSBM impacts, such as whether research contributes to the development of new clinical guidelines and methods for studying and treating infectious disease.

#### CQI enhancing performance evaluation and career development

Trainee feedback from the Fall 2016 mid-program interviews (*n* = 8) showed that trainees were satisfied with the quality and amount of informal feedback but wanted more formalized evaluation on their performance and progress. In response to this feedback, the RMTP Program Director started conducting annual performance reviews using a standardized evaluation form after each trainee’s research presentation. Trainee feedback from the exit interviews in 2018 (*n* = 4) indicated the annual committee meeting did provide a formal review, and this meeting was useful for evaluating their performance and progress. Building on this enhancement, the program also incorporated individualized career development plans (IDPs) to support trainees in exploring potential career pathways. Trainees worked collaboratively with their mentors and an oversight committee to develop and refine their IDPs, aligning their training activities with their long-term career goals. This addition not only formalized performance evaluations, but also provided structured guidance to help trainees identify and pursue careers in areas such as academia, industry, healthcare consulting and medical writing. Using an inclusive leadership approach, RMTP mentors and oversight committee members encouraged diverse perspectives and demonstrated value for trainees’ unique career interests. By the 2023 mid-point interviews (*n* = 8), seven of the eight trainees reported feeling confident in their growth toward becoming independent researchers and all eight trainees expressed progress toward feeling confident as they continued to work on activities such as submitting their first peer-reviewed publication or grant applications. All trainees expressed appreciation for the individualized career-focused support provided by the program.

#### CQI increasing diversity and inclusion

Applicant and awardee demographics from Fall 2015-Spring 2017 highlighted a lack of participation from underrepresented minorities, as defined by the NIH [i.e., people who identify being African American, Black, American Indian, Alaska Native, Native Hawaiian or other Pacific Islanders; Hispanic and/or Latino; women; having a disability; and/or those from disadvantaged backgrounds (e.g., first-generation college students, individuals from rural settings, or those with low socioeconomic status)] (44). In response, the RMTP Program Director enhanced recruitment strategies in Fall 2016. This plan included targeted outreach efforts to establish partnerships with minority-serving institutions and modifications to existing applications review criteria to prioritize a more holistic review. Additionally, the Emerging Scientific Scholars Program (ESSP) was introduced during the 2022 academic year to further enhance diversity at Boston University. The ESSP aimed to attract outstanding Ph.D. students from underrepresented groups by offering one-time financial scholarships. These scholarships were designed to support students transitioning to higher cost of living areas, such as Boston, and are tailored for United States citizens or permanent residents who are economically disadvantaged individuals, or first-generation college students. Scholarships are either included with the admission letter or communicated separately, with the primary objective of broadening the inclusivity of the incoming class. This financial support is part of an ongoing commitment to help students overcome economic barriers associated with relocating for advanced studies. Collectively, the changes implemented between 2015 and 2023 have contributed to notable increases in the representation of underrepresented groups in the program, as evidenced by demographic trends detailed in Table 3, as well as greater geographic diversity (data not shown).

**Table 3:** RMTP demographic data for awarded trainees 2015–2023 (*n* = 38).

Self-identified demographic categories	2015–2018 (*n* = 19)	2020–2023 (*n* = 19)
Women	58% (*n* = 11)	74% (*n* = 14)
Hispanic/Latino ethnicity	5% (*n* = 1)	26% (*n* = 5)
Racial underrepresented minority	0% (*n* = 0)	11% (*n* = 2)

Racial Underrepresented Minority categories include American Indian/Native Alaskan; Native Hawaiian or Other Pacific Islander; Black or African American.

#### TSBM clinical impact

Increasing the diversity of regenerative medicine research trainees aligns with our overall RMTP goals of increasing the overall regenerative medicine workforce (a broader TSBM clinical impact). Based on team science principles of inclusive leadership and creating a climate for inclusion, RMTP leaders partnered with our internal advisory board to collaboratively create the RMTP trainee recruitment plan following our WE-CQI process: collaborative planning, shared action, and team reflection and learning. This has allowed our RMTP program to successfully grow in all underrepresented demographic categories. By striving to increase the diversity of highly trained and competent researchers and practitioners in regenerative medicine, we aim to help enhance the larger regenerative medicine community. A more diverse workforce in this field has the potential to drive more innovative and novel research practices and theories. When there is a climate for inclusion, workforce diversity has shown to increase the generation of novel and groundbreaking ideas (45). When diverse members of a research community are treated in an inclusive manner, this creates a broader talent base and increases access to a wider range of knowledge, insights, and perspectives essential for innovation (45). This holds promise for enhancing health equity research topics and increasing participation of diverse and underrepresented participants in clinical trials. In essence, the down-stream health and societal benefits of such engagement can lead to the development of medical treatments that better serve underrepresented communities, ultimately reducing health disparities and improving outcomes for all populations.

An example of our commitment to meaningful impact within the TSBM clinical domain is our focus on equipping trainees with the skills and resources necessary to become independent researchers through their RMTP training, mentoring, and access to resources. This approach has proven successful in that 91% (*n* = 31) of our trainees remain engaged in regenerative medicine research fields in academia and industry, while three trainees remain engaged in healthcare consulting, medical writing, and health system clearance coordinating careers. Our trainees are making significant contributions across a variety of areas, including biomedical technologies, drug development, biological factors and products, software technologies, investigative procedures, diagnostic procedures, and therapeutic procedures. Table 4 provides specific examples of industry research initiatives where our graduated trainees are driving innovation and are making an impact.

**Table 4:** Examples of RMTP graduated trainee’s employer and clinical topic area.

RMTP trainee employer	Clinical topic area
Plaia Technologies	Artificial intelligence consulting services
Sarepta	Development of precision genetic medicines
Pyxis Oncology, Inc. (PYXS)	Development of antibody therapeutics to cancer
Invicro	Quantitative biomarkers, advanced analytics and imaging solutions
Takeda	Pharmaceutical
Satellite Bio	Solid organ cells as medicine
United Therapeutics	Creating products for chronic illnesses
GENEWIZ	Sequencing, gene synthesis
Lumanity	Commercial strategies consulting
Sanofi	Development of breakthrough medicines and vaccines

#### TSBM community and public health impact

One of our graduated trainee’s current career position is within CME Outfitters, LLC, an independent accredited provider of multidisciplinary continuing medical education & accreditation services. This entails striving to increase health education resources with the goal of helping medical professionals improve the quality and delivery of health care services. Part of this entails helping current researchers and academics translate their scientific findings for a broader community audience, providing manuscript writing and editing services for biology and related research fields. In essence, their work aims to provide engaging evidence-based content that resonates with community and public health audiences. The down-stream health and societal benefits would be increased accessibility of science with a focus on marginalized communities.

#### TSBM economic impact

In the context of the Boston University CTSA, the RMTP program has demonstrated significant economic benefits, particularly in fostering financial mobility and driving innovation through diversity and industry collaborations. By increasing the representation of individuals from underrepresented groups in regenerative medicine research, the program not only addresses inequities in representation but also provides pathways for wealth generation and career advancement. This financial mobility is supported through initiatives such as the ESSP, which provides scholarships to economically disadvantaged students, helping them transition into high-cost areas like Boston to pursue their education and careers. These efforts enable individuals from underrepresented backgrounds to access high-demand fields in biomedical research and industry, creating long-term economic benefits for their families and communities.

Beyond its impact on individual trainees, the RMTP has contributed significantly to innovation and translational science through patents and industry partnerships. TL1 trainees and faculty have been instrumental in securing key patents, such as U.S. Patent No. 10,975,357 B2, titled *Methods and Compositions Related to Differentiated Lung Cells*, which was co-invented by a TL1 trainee, Anjali Jacob. Additional patents include U.S. Patent No. 10,386,368 B2, *Isolation of Human Lung Progenitors Derived from Pluripotent Stem Cells*, Patent No. 10,590,392 B2, *Generation of Airway Epithelial Organoids from Human Pluripotent Stem Cells*, Patent No. 10,449,221 B2, *Differentiation of Stem Cells into Thyroid Tissue*, PCT Application No. PCT/US21/18714, *Generation of Airway Basal Stem Cells from Human Pluripotent Stem Cells*, Trackone Application No. 18/792,994, *Airway Basal Cell Engraftment Methods*, and US Patent Application No. 63/698,841, *Materials and Methods for the Derivation Lung-Specific Mesenchymal Progenitor Cells from Pluripotent Stem Cells*. These patents underscore the RMTP’s role in advancing regenerative medicine by addressing critical scientific and clinical challenges while creating intellectual property with significant commercial potential (see Table 5 for a description of impact of these patents).

**Table 5:** Example RMTP patents and TSBM impacts.

Patent title	U.S. patent #	Description	TSBM impact
Methods and compositions related to differentiated lung cells	10,975,357 B2	This patent enables the generation of lung cells derived from pluripotent stem cells, which are invaluable for modeling diseases such as pulmonary fibrosis and for high-throughput drug screening. By providing a reliable platform to study lung diseases, this technology has attracted interest from pharmaceutical companies aiming to develop targeted therapies, such as GSK in their collaboration with the CReM.	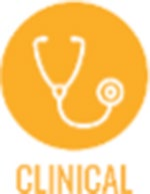 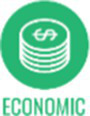
			New procedure for studying lung diseases and high-throughput drug screening; enables safe and cost-effective treatment development
Isolation of human lung progenitors derived from pluripotent stem cells	10,386,368 B2	This innovation provides a method to isolate lung progenitor cells, a critical step for regenerative therapies. These cells have potential applications in developing treatments for chronic respiratory conditions and in advancing cell-based transplantation therapies, which represent a rapidly growing sector in biotech.	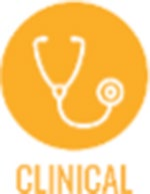
			New procedure for isolating lung progenitor cells
Generation of airway epithelial organoids	10,590,392 B2	Organoid technologies derived from this patent enable researchers to recreate functional airway tissue in vitro, which is crucial for studying infectious diseases such as COVID-19 and for testing novel therapeutics. This has significant commercial applications in both the pharmaceutical industry and precision medicine.	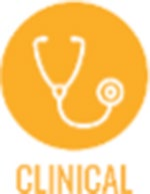 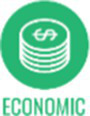
			New procedure for studying infectious diseases; enables safe and cost-effective treatment development
Differentiation of stem cells into thyroid tissue	10,449,221 B2	This patent focuses on generating thyroid tissue from stem cells, which could lead to novel therapies for thyroid disorders, including hypothyroidism. The ability to produce thyroid tissue in vitro also has implications for personalized medicine and drug testing, which could lead to licensing opportunities in the biotech sector.	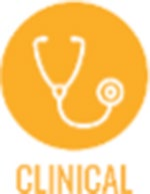 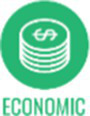
			New procedure for studying thyroid disorders; enables safe and cost-effective treatment development

TSBM icons: The Translational Science Benefits Model and Translating for Impact Toolkit^©^ 2017–2023, created by the Institute of Clinical and Translational Sciences at Washington University in St. Louis and available at *translationalsciencebenefitsmodel.wustl.edu*, is licensed under *Attribution-NonCommercial-ShareAlike 4.0 International (CC BY-NC-SA 4.0)*.

These patents underscore the RMTP’s role in advancing regenerative medicine and creating intellectual property with significant commercial potential. The program has also fostered strategic industry partnerships, notably the recently established collaboration in 2024 with GlakoSmithKline (GSK), a global biopharmaceutical leader. This partnership leverages the cutting-edge stem cell technology developed at CReM to advance the understanding and treatment of lung diseases, such as pulmonary fibrosis. Through this collaboration, GSK provides funding and expertise to scale up drug development efforts, translating basic research into potential therapies. Importantly, this collaboration also benefits RMTP trainees by offering them a unique opportunity to interact with industry professionals and gain insights into the biotech sector, where most graduate students ultimately pursue careers (thereby increasing economic mobility for members of the regenerative medicine workforce). Working alongside GSK during their training will afford trainees an opportunity to familiarize themselves with industry standards and processes, fostering career development and preparing them for future roles in the biotech and pharmaceutical industries. These interactions help trainees bridge the gap between academic research and industry application, enabling them to contribute effectively in both settings while conducting their dissertation research. This partnership not only enhances the RMTP’s translational impact but also demonstrates how academic-industry collaborations can drive economic and scientific progress.

These combined efforts highlight the RMTP’s multifaceted down-stream economic contributions: fostering financial mobility and equity for underrepresented individuals, generating valuable intellectual property, and advancing translational science through industry partnerships. More specifically, together, these outcomes illustrate how the RMTP serves as a model for leveraging diversity and innovation to create widespread economic and societal benefits.

#### TSBM policy impacts

Utilizing the Overton Index policy impact analysis tool (43), we searched 39 publications’ PMIDs/DOIs that were published by graduated RMTP trainees and included the citation for our Boston University CTSI TL1 grant (1TL1TR001410). Five publications (46–50) were cited in three policy documents (51–53) and two clinical guidelines (54, 55) by five policy sources across the United States, European Union, United Kingdom, and Canada. The three policy document publications cover the topics of replacing animal-based research models with human-relevant models in oncology and non-animal models in respiratory tract diseases. The two clinical guidelines cover the topics of clinical use of esophageal physiologic testing for diagnosing and managing esophageal disorders and venous thromboembolism prophylaxis in patients undergoing total hip or knee replacement surgeries. The down-stream health and societal benefits include setting formal quality assurance standards for various health study approaches and treatments.

## Discussion

CTSA evaluation teams are challenged with implementing CQI processes that enhance the organization and infrastructure of CTSA programs, along with executing evaluation activities that assess health and societal impact of translational science. Limited theory and evidence-based approaches exist that help evaluation teams simultaneously accomplish both CQI and impact evaluation. Drawing on impact evaluation research from the TSBM (5), CQI (6–8, 16), and team science (9–12), we develop a theory-driven, team-based approach to CQI and impact evaluation. We extend the TSBM logic model framework to incorporate CQI and evaluation activities, providing a practical approach other CTSA evaluation teams can use to guide their efforts.

Our team science-based approach to CQI and evaluation involves creating a climate for inclusion wherein all program members feel like important members of the group and that their unique talents and perspectives are appreciated. This is accomplished through CTSA leaders (i.e., evaluation and program leaders) demonstrating inclusive leadership behaviors. Several published articles provide examples of how leaders and evaluators can foster inclusive leadership (25, 29, 56–58), and thereby inclusion and psychological safety. A brief review of this literature suggests leaders who recognize that every group member has unique needs and abilities, expresses appreciation for group members’ unique talents and abilities, and values the contributions of others helps foster a climate for inclusion (25). In addition, when leaders proactively seek and value feedback from group members, regardless of group members’ job positions or titles, this helps engender feelings of inclusion within the group (9, 27). This is particularly important when designing CTSA program CQI and evaluation activities as every program member needs to feel a shared sense of responsibility in the creation and implementation of improvement and evaluation efforts. One way this can be achieved is through inviting each program member to partner in the development of CQI and evaluation activities. In our theory-based CQI and evaluation approach we collaboratively create a logic model which provides a road map and guideline for how CQI and evaluation activities are embedded within program activities and ultimately how the program strives to make health and societal benefits. The success of the WE-CQI logic model and creating a shared sense of responsibility for the implementation of the activities in the logic model is largely dependent upon the creation of a climate for inclusion, otherwise this exercise fails to meaningfully engage CTSA members. When program members are not engaged in the CQI and evaluation process, implementation of these activities are weakened, resulting in low-quality program fidelity, data collection, and team reflection, learning, and improvement.

While previous research has demonstrated promising interventions for fostering inclusion (25, 29, 56–58) and psychological safety (59), more research is needed on how these approaches enhance outcomes for CQI and evaluation processes within complex multi-team systems. We strive to help address this gap in the literature using case study methods and illustrating how the WE-CQI team-science based approach can enhance quality improvement and evaluation activities. Through mixed-methods data collection, results highlighted how WE-CQI efforts helped strengthen several aspects of the RMTP (e.g., increasing diversity representation among trainees) and how RMTP activities ultimately made significant health and societal benefits using the TSBM framework. For example, several RMTP activities have led to the development of new tools and procedures for safely and effectively studying lung and thyroid diseases, which has enabled a more rapid and efficient process for creating novel treatments. This aligns with the mission of NCATS in the ability to effectively turn research into health solutions more quickly (60). As CTSA evaluation teams strive to help programs continuously improve through team science-based approaches to CQI, this can help increase the likelihood that program resources and activities get better at achieving meaningful TSBM impacts. Other CTSAs can apply our developed logic model and CQI and evaluation framework as a practical guide for how to enhance CTSA activities and the quality and impact of translational science. Having CTSA leaders and evaluators trained in inclusive leadership approaches and on the importance of fostering inclusive and psychologically safe team climates may be essential to successfully implementing a team-science based approach to CQI and evaluation activities.

### Limitations

Future research is needed to test the generalizability of this approach to other CTSA programs. It is possible that variation exists in the fidelity of implementing the WE-CQI framework given the level of expertise and ability of CTSA evaluators in using a team science approach in the collaborative planning and reflection and learning phases of the model. For example, CTSA evaluators well-skilled in inclusive leadership and fostering a climate for inclusion may yield stronger results to successfully implementing the framework; however, while there is theoretical evidence and case study methods to support this hypothesis, more empirical research is needed. Current research on evidence-based inclusive leadership trainings and interventions is limited and need further development. Likewise, additional leadership approaches, such as authentic leadership and leader-member exchange (61, 62), could be examined as alternative avenues for implementing the WE-CQI framework.

## Conclusion

CTSA evaluation teams face the dual challenge of implementing CQI processes designed to enhance the effectiveness of CTSA programs, while simultaneously evaluating the health and societal impacts of their initiatives. Building on research from CQI, impact evaluation and the TSBM framework, and team science, we have developed a theory-driven, team-based logic model that integrates CQI and evaluation processes. Using a case study of our CTSA supported programs (TL1 Regenerative Medicine Training Program), we demonstrate the practical application of this approach by providing a concrete example of how the WE-CQI framework can be utilized to support CQI and impact evaluation. Our findings offer other CTSA programs with a replicable and adaptable framework to guide their evaluation teams in striving for excellence in clinical and translational science. By adopting this approach, evaluation teams can better align their efforts with translational science goals, ensuring meaningful contributions to the greater public health.

## Data Availability

The datasets presented in this article are not readily available because participant confidentiality. Requests to access the datasets should be directed to Kim Brimhall brimhall@bu.edu.
